# Genetic Diversity of Microcystin Producers (Cyanobacteria) and Microcystin Congeners in Aquatic Resources across Africa: A Review Paper

**DOI:** 10.3390/toxics10120772

**Published:** 2022-12-10

**Authors:** Mathias Ahii Chia, Ilu Ameh, Korie Chibuike George, Emmanuel Oluwadare Balogun, Suwebat Ayanronke Akinyemi, Adriana Sturion Lorenzi

**Affiliations:** 1 Department of Botany, Ahmadu Bello University, Zaria 810107, Nigeria; 2 Department of Biochemistry, Ahmadu Bello University, Zaria 810107, Nigeria; 3 African Centre of Excellence for Neglected Tropical Diseases and Forensic Biotechnology, Ahmadu Bello University, Zaria 810107, Nigeria; 4 Department of Plant Biology, Osun State University, Osogbo 210001, Nigeria; 5 Department of Cellular Biology, Institute of Biological Sciences, University of Brasília—UnB, Brasília 70910-900, Brazil

**Keywords:** biological risk, cyanobacteria, cyanotoxin, water supply, African continent, molecular markers, *mcy* genes

## Abstract

Microcystins are produced by multifaceted organisms called cyanobacteria, which are integral to Africa’s freshwater environments. The excessive proliferation of cyanobacteria caused by rising temperature and eutrophication leads to the production and release of copious amounts of microcystins, requiring critical management and control approaches to prevent the adverse environmental and public health problems associated with these bioactive metabolites. Despite hypotheses reported to explain the phylogeography and mechanisms responsible for cyanobacterial blooms in aquatic water bodies, many aspects are scarcely understood in Africa due to the paucity of investigations and lack of uniformity of experimental methods. Due to a lack of information and large-scale studies, cyanobacteria occurrence and genetic diversity are seldom reported in African aquatic ecosystems. This review covers the diversity and geographical distribution of potential microcystin-producing and non-microcystin-producing cyanobacterial taxa in Africa. Molecular analyses using housekeeping genes (e.g., 16S rRNA, ITS, *rpoC1,* etc.) revealed significant sequence divergence across several cyanobacterial strains from East, North, West, and South Africa, but the lack of uniformity in molecular markers employed made continent-wise phylogenetic comparisons impossible. *Planktothrix agardhii, Microcystis aeruginosa,* and *Cylindrospermopsis raciborskii* (presently known as *Raphidiopsis raciborskii*) were the most commonly reported genera. Potential microcystin (MCs)-producing cyanobacteria were detected using *mcy* genes, and several microcystin congeners were recorded. Studying cyanobacteria species from the African continent is urgent to effectively safeguard public and environmental health because more than 80% of the continent has no data on these important microorganisms and their bioactive secondary metabolites.

## 1. Introduction

The 3.5 billion-year-old phylum of photosynthetic prokaryotes known as cyanobacteria can be found in various habitats, such as desert rocks, freshwater bodies, hydrothermal vents, marine ecosystems, and Antarctic lakes [1]. Eutrophication, increased solar irradiation, and high temperatures from global warming promote the excessive proliferation of harmful cyanobacteria in aquatic systems [2]. Some cyanobacterial strains synthesize bioactive secondary metabolites (e.g., cyanotoxins), which could adversely affect human and ecosystem health [3]. For example, animal and human mortality have been recorded following exposure to significant doses of some cyanotoxins [4]. Cyanotoxins vary in toxicity and chemical structure, falling into three major chemical structures: lipopolysaccharides, cyclic peptides, and alkaloids [5].

There is a lack of information on the occurrence of cyanobacteria blooms in African aquatic resources. A UNESCO survey found that, except in Ethiopia, Kenya, Morocco, and Southern Africa, knowledge of the events of cyanobacterial blooms in Africa is almost non-existent [6,7]. In addition, very little has been reported about the underlining morphology, genetic characterization, and geographical origin among cyanobacterial strains across Africa. This knowledge gap limits our understanding of cyanobacteria distribution, diversity, toxin production, and the nature of their congeners, emphasizing the risk of exposure via contamination of potable water resources. To comprehend cyanobacteria’s phylogeny, geographic origin, and distribution in Africa, an evolutionary reconstruction of previous relationships using genetic material (DNA and RNA) or protein sequences, is necessary to close this information gap. Numerous phylogenetic-based scientific hypotheses contend that speciation, extinction, and dispersal can account for the development of biogeographic patterns [8]. 

**Figure 1:**
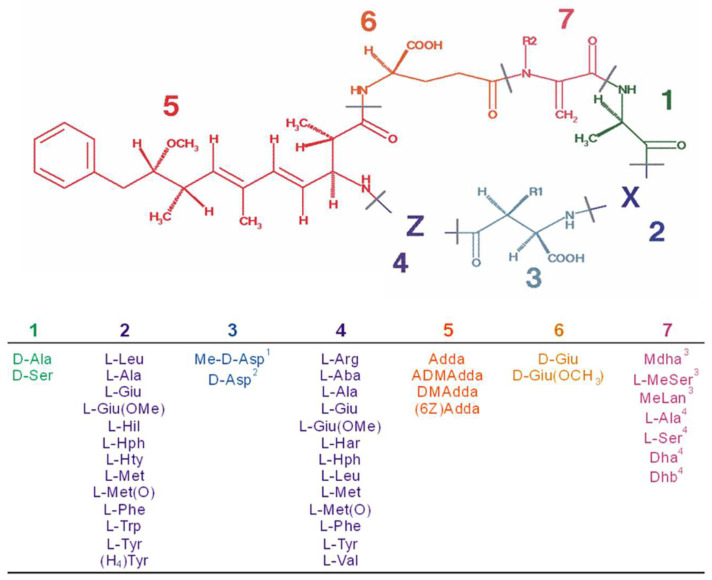
Microcystin chemical structure generalized as cyclo-D-Ala^1^-X^2^-D-MeAsp^3^-Z^4^-Adda^5^-D-Glu^6^-Mdha^7^, where X and Z denote the highly variable L-amino acids present at the second and fourth positions, with possible different combinations of seven amino acids that can produce different microcystin variants (modified from Butler et al. [13]).

**Figure 2:**
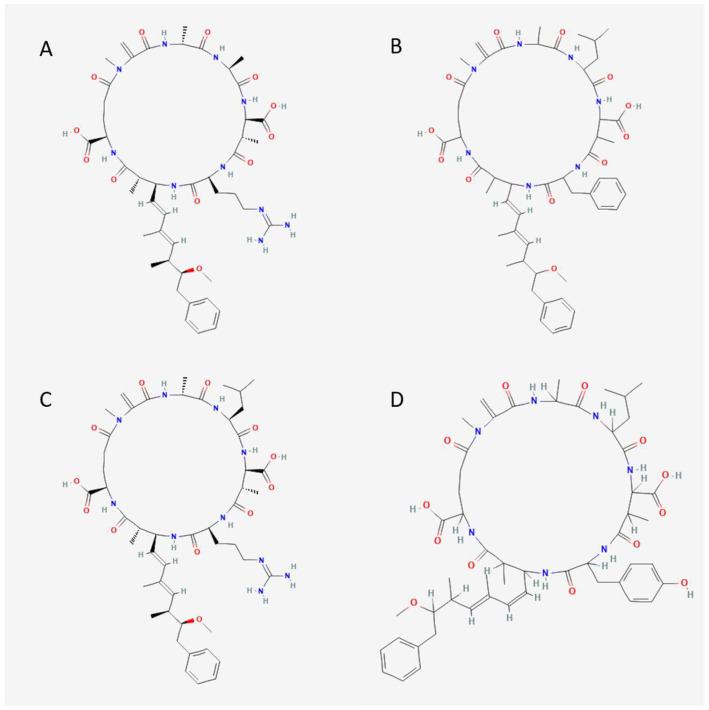
Microcystin congeners. (**A**) Microcystin-AR, (**B**) Microcystin-LF, (**C**) Microcystin-LR, (**D**) Microcystin –LY. Source: PubChem.

In freshwater and brackish waterbodies worldwide, different cyanobacteria synthesize a class of small bioactive peptides known as microcystins [9]. Microcystins are cyclic heptapeptides with the same unusual amino acid known as ADDA (3-amino-9methoxy-2,6,8-trimethyl-10-phenyldeca-4,6-dienoic acid) that forms part of the general cyclo-(-d-Ala-l-X-d-MeAsp-l-Z-Adda-d-Glu-Mdha) structure (Figure 1). The d-MeAsp is d-erythro-b-methylaspartic acid, Mdha is *N*-methyldehydroalanine, and X and Z are variable l-amino acids [10]. Changes in the variable amino acids X and Z result in over 300 structural microcystin congeners (Figure 2) [11]. Microcystin congeners are named using the one-letter abbreviation for the amino acids substituted at the X and Z positions, respectively (Figure 1). This can be seen on the depicted analogs/congeners (A) Microcystin-AR, (B) Microcystin-LF, (C) Microcystin-LR, (D) Microcystin –LY (Figure 2), and on any of the identified congeners. Despite their environmental importance, microcystin distribution, isoforms, and levels have been scantily investigated in African water bodies [12]. This review aims to highlight existing information regarding cyanobacteria’s morphological, genetic, and chemical diversity in water bodies across the East, West, North, and South of Africa. The reviewed data cover field samples and isolated strains morphologically examined and subjected to a DNA-based molecular analysis to reveal genetic variation and geographical divergences or relatedness of cyanobacteria. In addition, we provide a compilation of microcystin detection (molecular and chemical methods) and detected concentrations in African water bodies as a platform for future studies.

## 2. Toxicosis of Microcystins in Africa

The bioactivity of microcystins is based on the inhibition of the catalytic subunits on serine-threonine protein phosphatases 1 and 2A due to the covalent binding of microcystins to these enzymes [14,15,16], which causes the hyper-phosphorylation of cytoskeleton-associated proteins in hepatocytes. This rapid breakdown of the sinusoidal architecture and cell attachment is followed by a disruption of cytoskeletal compartments and a subsequent rearrangement of filamentous actin [17,18]. Low doses of exposure to microcystin cause chronic inflammation, focal degeneration of hepatocytes, and metabolite accumulation, which may lead to progressive kidney and liver failure. Higher doses of microcystin result in immediate intrahepatic hemorrhage, liver necrosis, and hypovolemic shock. Other effects of microcystin exposure include alterations in mitochondrial function, alterations in intracellular calcium levels, free radical formation, and oxidative stress, all of which could contribute to apoptosis and hepatotoxicity. In addition to being hepatotoxic, microcystins also harm exposed organisms’ ability to reproduce and develop, and induce nephrotoxicity, cardiovascular disease, immunomodulation, endocrine disturbance, and gastrointestinal issues [19]. 

Although there are no documented human deaths due to microcystin poisoning in Africa, direct contact with high concentrations of microcystins has been reported to cause adverse reactions. These include eye and skin irritations, gastrointestinal distress, nausea, and human liver toxicity [20]. Hepatotoxicosis has been reported in domestic animals after exposure to microcystins [21]. Microcystins partly caused the mass mortalities of Lesser Flamingos in Tanzania and Kenya. This conclusion was made after a histopathological analysis of liver tissues [22,23]. Furthermore, several other deaths of wild animals, such as rhinoceroses, zebras, and wildebeests have been associated with a daily intake of MC-LR ranging from 754.29 to 2566.6 µg Kg^−1^ day^−1^ in South Africa [24]. 

## 3. The Taxonomic Diversity of African Cyanobacteria Using Genetic Markers

Most research on the genetic diversity of cyanobacteria in Africa has focused on toxin-producing versus non-toxin-producing strains that occur simultaneously or consecutively throughout bloom seasons [25,26]. In addition, the identification of cyanobacteria in Africa has traditionally been made microscopically based on morphological differences between field and isolated species/strains. A compilation of geographic and molecular information from cyanobacterial studies in Africa is summarized in Table 1. 

Advances in molecular biology have facilitated the use of polyphasic approaches involving chemotaxonomic, phenotypic, and genotypic data to characterize cyanobacteria. Genotypic diversity through PCR (polymerase chain reaction) amplification of target sequences, cloning, and DNA sequencing of isolated strains and field samples has been poorly described in Africa [27,28,29]. The most commonly used genetic markers in Africa include 16S rRNA, PC-IGS, 16S-23S ITS1-L, 16S-23S ITS1-S, *rpo*B, *rpo*C1, and 16S-23S ITS. These molecular markers have been employed to understand the taxonomy and phylogeography of cyanobacteria worldwide [8,30].

**Table 1:** Compilation of geographic and molecular information from African cyanobacterial studies.

Sub-Region	Country/Sampling Sites	Identified/Isolated Species	Number of Strains/Sequences Analyzed	Method of Identification	Taxonomic Genetic Marker and/or Molecular Technique	Microcystin Detection—Molecular Markers	Microcystin Detection—Chemical Analysis	Location of Closer Related spp. /Genotype/Strain	Reference
North Africa	Wadi El-Natrun, Egypt	*Spirulina, Oscillatoria, Microcystis*	NA	SEM, Light microscopy	16S rDNA, SDS-PAGE	28 ^a^	NA	NA	[28]
	Tunisia	*Microcystis aeruginosa, Raphidiopsis raciborskii, Planktothrix agardhii*	27	NA	16S–23S rRNA ITS, *rpo*C1	NA	NA	South Africa, Sweden, and USA strains. Toxic Micro-HJ-02 strain clustered with a French strain	[31]
	BirM’cherga reservoir, Tunisia	*Raphidiopsis raciborskii*	4	NA	16S rRNA, *rpo*C1	*mcy*A, *mcy*E	HPLC and MALDI-TOF	Distinct from other African strains	[25]
East Africa	Lake Bogori, Kenya	*Arthrospira fusiformis, Anabaenopsis, Synechococcus, Arthrospira, Spirulina, Oscillatoria, Leptolyngbya*	NA	Light microscopy	Multilocus 454- sequencing (16S rRNA and (*cpc*BA-IGS, Metagenomics	NA	NA	NA	[29,32]
	Kenya, Tanzania, Uganda	*Arthrospira*	18	Width of trichome, types of helix, and apical shape of trichomes	16S-23S ITS (31 *), *cpc*BA-IGS (23 *)	NA	NA	India, Asia	[33]
	Kenya, Uganda	*Microcystis* *aeruginosa*	17 (8)	Light microscopy	PC-IGS, ITS1 rDNA	4 (3 MC producing)	NA	Australia, Brazil	[34]
	Uganda	*Raphidiopsis raciborskii*	(44 **)	Morphological traits	ITS1, PC-IGS, *nif*H, *rpo*C1	NA	NA	Germany	[35]
	Sediments and plankton of saline-alkaline and freshwater lakes of Kenya	*Anabaenopsis*, *Umezakia*, *Arthrospira* sp., *Chroococcidiopsis* sp.	Nucleotide sequences (8) from DGGE bands: Nostocales (5), Pleurocapsales (1), Oscillatoriales (2)	BLAST search analysis	PCR-based denaturing gradient gel electrophoresis (DGGE) analysis of 16S rRNA gene fragments	*mcy*E and *nda*F	NA	Kenya	[33]
	Ugandan freshwaters	*Anabaena*, *Aphanocapsa*, *Chroococcus*, *Merismopedia*, *Microcystis*, *Planktolyngbia*, and *Pseudanabaena*	NA	Morphological keys	PC-IGS	*mcy*B and *mcy*E	HPLC-DAD, MALDI-TOF MS, Q-TOF	Uganda	[36]
West Africa	Rivers, lagoons, and coastal waters, Nigeria	*Microcystis aeruginosa,* *Planktothrix aghardii,* *Anabaena* sp.	NA	Light microscopy	NA	*mcy*E	Abraxis microcystins /Nodularin Adda Kit (Abraxis Inc., Warminster, PA, USA)	Southern Germany, Korea, Japan	[12]
	Freshwater lakes and reservoir, Senegal	*Raphidiopsis raciborskii, Raphidiopsis africana* *Raphidiopsis* sp.	18	Light microscopy, the ability to differentiate heterocysts when grown in a nitrogen-free medium	16S rRNA, ITS1-S, ITS1-L, *rpo*Cl, *nif*H [10 *]	NA	NA	America, Europe	[27]
Southern Africa	Hartbeespoort Dam, South Africa	*Sphaerospermopsis reniformis, Sphaerospermopsis aphanizomenoides,* *Raphidiopsis curvispora, Raphidiopsis curvata, Raphidiopsis mediterrranea, Microcystis aeruginosa*	27 (1 MC producing)	Light microscopy	NA	*mcy*E 41 ^b^ (35) MC (1 ^d^; MC-AnaR)	NA	Tropical and subtropical regions of Africa	[26]
	Crusts Karoo and Nama Karoo, South Africa	Consortia of cyanobacteria	45	Morphological features	16S rRNA (140 *)	103 ^a^ (11 ^d^)	NA	NA	[37]
	South Africa	*Microcystis aeruginosa*	23	Light microscopy	AFLPs	NA	NA	Japan, Europe, North America	[38]
	Theewaterskloof Dam, South Africa	*Anabaena* *ucrainica*	NA	SEM	16S rRNA	NA	NA	NA	[39]
	Phakalane ponds, Botswana	*Microcystis novacekii*	NA	Microscopic identification BLAST search analysis	16S rRNA	*mcy*A, -B, -C, -D, -E and –G	LC-ESI-MS and solid-phase extraction (SPE) steps	Botswana	[40]

NA: not applicable; ^a^ Strain; ^b^ Variant; ^d^ Novel variant; MC: *Microcystis*; *: reference sequences; **: reference strains.

### 3.1. 16S Ribosomal RNA and 16S–23S rDNA Internal Transcribed Spacer (ITS)

The small subunit ribosomal RNA molecules of ribosomes, which are encoded by the 16S rRNA gene, are necessary for the process of translating genetic instructions into functional cell components via the translation of mRNA to proteins. Because it is a part of all self-replicating systems, ribosomal RNA is very easy to extract, and its slow rate of sequence change over time makes it possible to identify relationships between quite different species [41]. The 16S rRNA gene’s sequence was discovered to contain both conserved and changeable sections through a sequence analysis and structural modeling. Variable nucleotides appear at the surface of the ribosome, where it is hypothesized that nucleotide substitutions do not alter ribosome activity, in contrast to evolutionarily conserved nucleotides that appear toward the center of the ribosome at functional regions [42].

**Figure 3:**
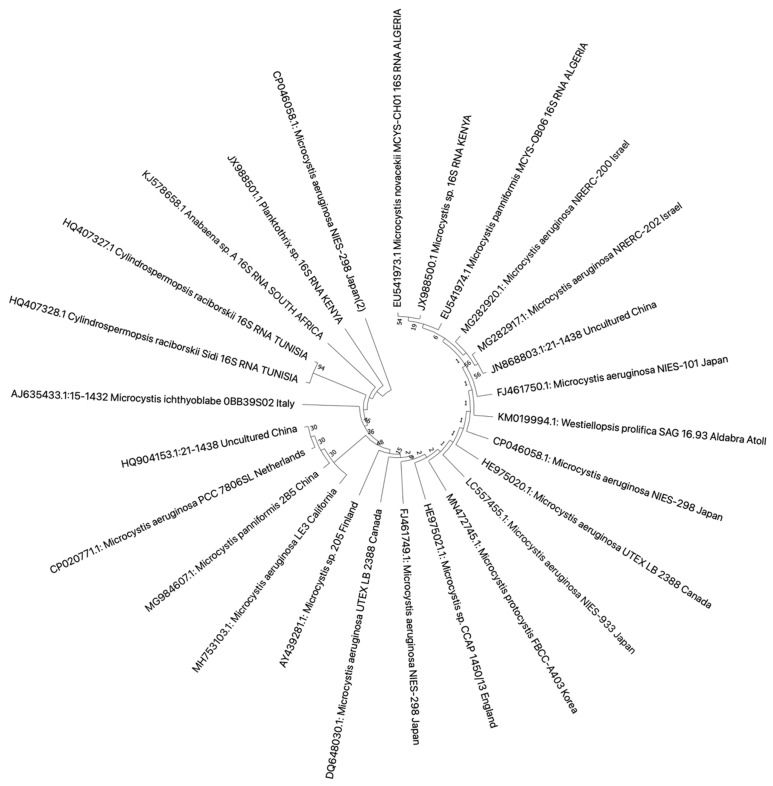
Neighbor-joining phylogenetic tree of the 16S rDNA gene of bloom samples and isolated cyanobacteria from Africa and other continents. The bootstrap consensus tree was inferred from 1000 replicates, and the evolutionary distances were computed using the maximum composite likelihood method. All ambiguous positions were removed for each sequence pair (pairwise deletion option). There were a total of 1462 bp in the final dataset. Evolutionary analyses were conducted in MEGA X version 11 for macOS.

**Figure 4:**
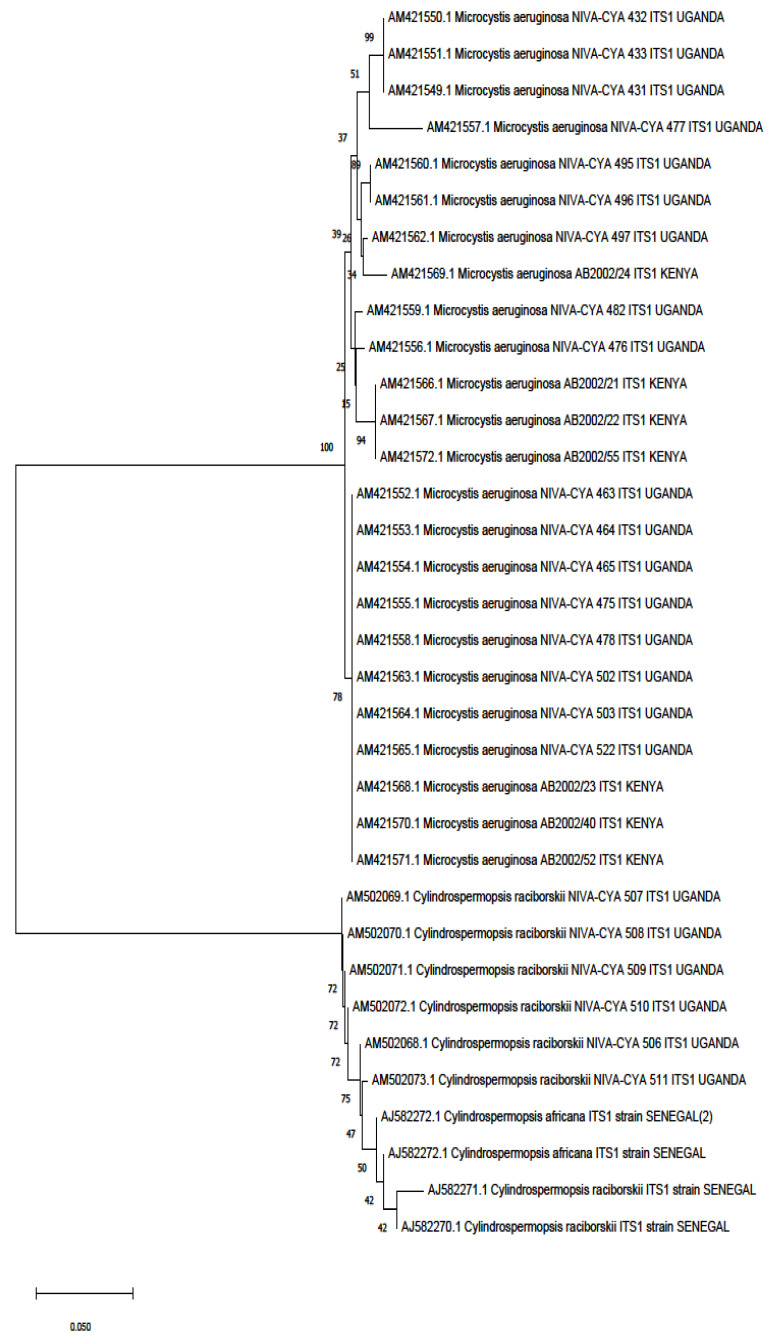
Phylogenetic tree constructed using the neighbor-joining method of the ITS1 sequences’ gene of cyanobacteria detected from studies on the continent. Branches corresponding to partitions reproduced in less than 50% of bootstrap replicates are collapsed. The percentage of replicate trees in which the associated taxa clustered together in the bootstrap test (1000 replicates) are shown next to the branches. The evolutionary distances were computed using the Kimura 2-parameter method and are in the units of the number of base substitutions per site. This analysis involved 34 nucleotide sequences. All ambiguous positions were removed for each sequence pair (pairwise deletion option). There was a total of 539 bp in the final dataset. Evolutionary analyses were conducted in MEGA X version 11.

In this review, we retrieved 16S rRNA and ITS1 (Figure 3 and Figure 4) sequences deposited in the National Center for Biotechnology Information (NCBI) database that had a study or data on African cyanobacteria strains to generate phylogenetic trees using the NJ (neighbor-joining) algorithm: a widely used method for a long time for constructing phylogenetic trees. NJ is a greedy algorithm based on the distance between sequences that endeavors to minimize the sum of all the branch lengths of the resulting tree. It is out of the scope of our paper to cover the limitations or comparisons of methods employed for a DNA sequence analysis. Moreover, all the figures (Figure 3 and Figure 4) contain data from annotated sequences of cyanobacteria blooms and isolated strains in Africa and other continents in the NCBI database. There was a tendency for 16S rRNA sequences of isolated strains from the same country to group independently of the cyanobacterial genera (Figure 3). Furthermore, cyanobacteria samples collected from Kenya and Algeria formed subclusters with cultured *Microcystis aeruginosa* and uncultured cyanobacteria from Israel and China, respectively (Figure 3). Another subcluster was formed by cyanobacteria isolated from Tunisia, South Africa, Kenya, and Japan. 

The combination of molecular markers increases the accuracy of the taxonomic classification of cyanobacteria. Fathalli et al. [25] reported using 16S rRNA and *rpo*C1 molecular markers to characterize four *Raphidiopsis raciborskii* strains isolated from Tunisia. The *rpo*C1 gene sequence was used to determine the phylogenetic connections among the strains, which were taxonomically identified using 16S rRNA sequences. The composite molecular markers revealed that Tunisian strains established a unique clade from other African strains included in the phylogenetic analysis. This result demonstrated that *rpo*C1 sequences can discriminate between close taxa—unfortunately, only a few studies have used this molecular marker in Africa. This strongly corroborates with previous findings showing that the *rpo*C1 gene sequence is extra selective at the species level compared to the 16S rRNA sequence [43]. Furthermore, the maximum likelihood (ML) phylogenetic tree of *R. raciborskii* worldwide strains inferred from combined sequences of 16S rRNA, ITS-L, ITS-S, and *rpo*C1 encompassing~2927 bp grouped into three well-supported distinct clusters: the European (I), African/American (II), and Asian/Australian (III) strains [31]. The African strains consisted of Tunisian strains. In a review article, Padisák [44] postulated that *R. raciborskii* originated in Africa. Over the years, it has spread to equatorial regions such as Indonesia and Central America, with a second radiation center in Australia, accounting for the more recent cyanobacteria invasion in the tropical, subtropical, and temperate regions. The distinct clustering of African–Australian *R. raciborskii* strains supports this evidence [27,45].

Using the Denaturing Gradient Gel Electrophoresis (DGGE) technique coupled with the 16S–23S rDNA internal transcribed spacer (ITS), van Gremberghe et al. [46] analyzed the local and regional genetic diversity as well as the structure of *Microcystis* populations in 30 man-made reservoirs from Tigray (Northern Ethiopia) in wet and dry seasons. Both regional and local *Microcystis* ITS diversity (number of distinct ITS types in the DGGE profile per sample) were relatively low, with several dense blooms containing only a single ITS type (EG07). In addition, the authors observed a geographic signature and a limited influence of local environmental conditions on the *Microcystis* population structure, represented by the presence or absence of ITS types in the DGGE profile per sample and their relative abundances. Members of the genus *Microcystis* are the most investigated cyanobacteria worldwide, and Africa is no exception. Research by Moreira et al. [47] suggested that *Microcystis aeruginosa* originated on the African continent, with further Europe-mediated global dispersal of the species.

### 3.2. Amplified Fragment Length Polymorphism

Amplified fragment length polymorphism (AFLP), a PCR-based fingerprinting technique, and SDS-PAGE (sodium dodecyl sulfate-polyacrylamide gel electrophoresis), a protein profile analysis technique, have been used to characterize cyanobacteria in Africa molecularly. The SDS-PAGE protein profile was used for the first time to analyze the genus *Merismopedia* in Egypt (North Africa) [48]. Oberholster et al. [38] employed AFLP fingerprinting to highlight the differences among 23 strains of geographically unrelated *Microcystis* isolated from South Africa, and the UPGMA (unweighted pair group method with arithmetic mean) hierarchical clustering method revealed four groups. The NIES strains of Japanese origin clustered in Group 1; the European strains clustered in Group 2; the South African strains (northern SA) formed the third distinct group; and the strains collected from the central and southern regions of SA clustered together with the US strains in Group 4. Their results revealed extensive evidence of the applicability of AFLP in cyanobacterial characterization, validating its discriminative power towards the differentiation of unrelated *Microcystis* strains of the same species (*M. aeruginosa*), as well as highlighting the potential of genomic restriction fingerprinting in evolutionary studies. 

### 3.3. Omics—Metagenomics for Characterization of Cyanobacteria in Africa

Metagenomic studies of cyanobacteria are scarcely conducted in Africa. Using multilocus 454-amplicons sequencing, Dadheech et al. [32] used metagenomics to investigate the cyanobacterial community structure of Lake Bogoria, Kenya. A high degree of endemism was found for Oscillatoriales phylotypes (*Leptolyngbya*, *Spirulina*, *Oscillatoria*-like, and *Planktothricoides*) in the Bogoria hot springs. Different clades of *Synechococcus* represented Chroococcales. The pelagic zone and the sediments were inhabited by only a few taxa, dominated by *Arthrospira* and *Anabaenopsis*. *Arthrospira,* the main food for Lesser Flamingos, was detected in all three habitats (hot springs, pelagic zone, and sediment), indicating its resilience and critical role as a primary producer. We look forward to seeing additional metagenomics studies in Africa because taxonomic classification using this approach enables the establishment of the exact origin of species and cataloging or classifying various cyanobacterial groups inhabiting unique environments in Africa. 

## 4. Detection of Potentially African Microcystin-Producing Cyanobacteria by Molecular Markers

As previously stated, microcystins are a family of cyclic peptide toxins produced by cyanobacteria that are widely responsible for the toxicosis and death of wild and domestic animals worldwide due to their hepatotoxic effects. Microcystins are assembled on megadalton enzyme complexes consisting of a combination of nonribosomal peptide synthetases (NRPS), type I polyketide synthases (PKS-I), hybrid NRPS/PKS-I, and tailoring enzymes [9,49,50,51]. The proposed functions of proteins encoded by the microcystins’ biosynthetic gene cluster (*mcy*) are summarized in Table 2. 

**Figure 5:**
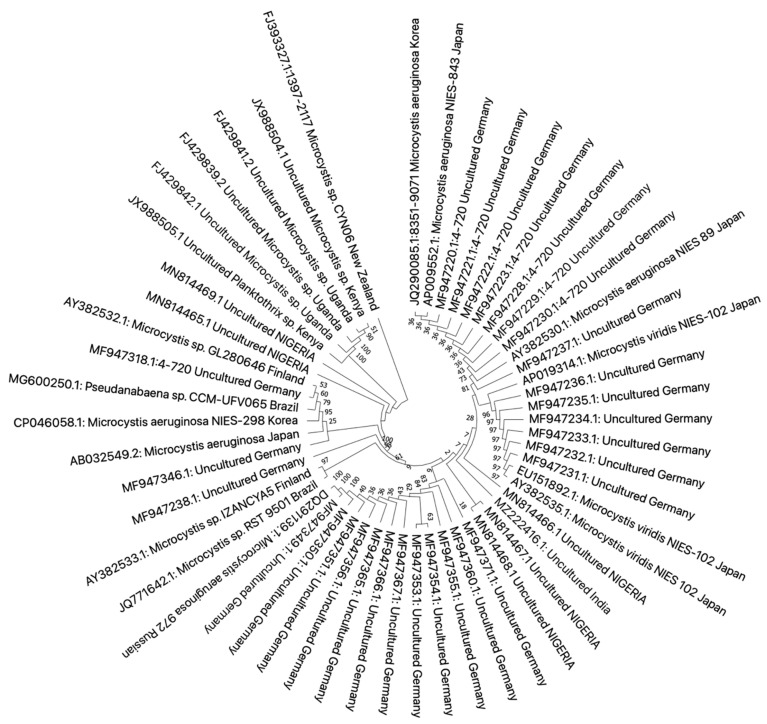
UPGMA phylogenetic tree of the *mcy*E gene of blooms and isolated cyanobacteria from Africa and other continents. The bootstrap consensus tree was inferred from 1000 replicates, and the evolutionary distances were computed using the maximum composite likelihood method. The analysis involved 54 nucleotide sequences. All ambiguous positions were removed for each sequence pair (pairwise deletion option). There was a total of 847 bp in the final dataset. Evolutionary analyses were conducted in MEGA X version 11 for macOS.

A pairwise comparison of *mcy*E gene sequences of samples collected from coastal areas of Nigeria showed identities higher than 86% (query coverage > 96%) with toxic *Microcystis* strains [12]. Most Nigerian *mcy*E sequences formed distinct clusters from those of other countries in Africa and other continents (Figure 5). Additionally, *mcy*E sequences of samples collected from Kenya and Uganda formed a distinct subcluster from other African samples and those obtained from other continents. PCR amplification and sequencing of the *mcy*A gene in samples collected from Hartbeespoort Dam, South Africa, revealed the presence of toxin-producing strains of *M. aeruginosa* in the dam [53]. 

Five other *mcy* genes (*mcy*BCDEG) were also PCR amplified and analyzed during this study. Integrating phylogeny, geographic niche partitioning, and analysis of the *mcy* gene cluster composition in bloom-forming *Planktothrix,* Kurmayer et al. [54] analyzed the genotypic dispersal of 138 strains from Europe, Russia, North America, and East Africa. Although the strains had at least remnants of the *mcy* gene cluster, various phylogenetic lineages might have evolved and adapted to specific ecological niches. Douma et al. [55] reported the presence of potentially toxic *Microcystis* strains in two Middle Atlas Mountains’ natural lakes in Morocco for the first time. A whole-cell multiplex PCR detected *mcy* genes, which allowed the simultaneous amplification of DNA sequences corresponding to specific *mcy* regions. 

Fathalli et al. [56] characterized 27 potentially toxic cyanobacteria strains, mainly of *M. aeruginosa*, *C. raciborskii,* and *P. agardhii,* from seven reservoirs in Northern and Central Tunisia. In most *Microcystis* isolates, six distinct segments of the microcystin synthetase *mcy* cluster (*mcy*A, -B, -C, -D, -E, and -G) were detected. The presence of a toxic strain of *M. aeruginosa* in the upper littoral zone of Lake Krugersdrift, South Africa, was accessed by detecting the *mcy*B and *mcy*D genes and confirmed through the use of ELISA [57]. This information is indexed in PubMed^®^ from the National Center for Biotechnology Information (NCBI) database and represents the toxic cyanobacteria molecular studies conducted in Africa. Further studies from the African continent are summarized in Table 1, indicating that toxin-producing cyanobacteria in African waters are of a public and environmental health concern. Several studies could not be included in the phylogenetic tree for *mcyE* (Figure 5) because the sequence accession numbers were not provided. 

## 5. Cyanobacterial Abundance and Microcystin Congeners Detected in African Waters

The abundance of cyanobacteria, microcystin congeners, and detected concentrations in several countries of the African continent are presented in Table 3. Here, we revised important aspects of cyanobacterial abundance and microcystin congeners recorded in African waters per region (East Africa, North Africa, South Africa, and West Africa, respectively).

### 5.1. East Africa

Using a polyphasic approach to characterize *M. aeruginosa* isolates from East African aquatic ecosystems, Haande et al. [34] reported ten chemotypes by a MALDI-TOF-MS oligopeptide analysis. Interestingly, only about 17% of the isolated strains produced microcystins, while most strains were found to produce the protease inhibitor oligopeptides aeruginosin and cyanopeptolin in freshwater and saline lakes. 

The phytoplankton composition of Ugandan freshwater lakes is similar to that found in other East African lakes, where it is common to find cyanobacteria as the dominant group. Although Ugandan lakes have different trophic conditions ranging from mesotrophic to hypertrophic, these conditions, coupled with the warm climate of East Africa, facilitate the excessive proliferation of cyanobacteria. *Anabaena* spp. and *Microcystis* spp. are the most frequent and dominant species found in eutrophic water bodies, with total microcystins concentrations ranging from 0.03 to 144 fg cell^−1^ and 0.02 to 10 μg MC-LR eq./L [36,86,87,88] (Table 3). The key congeners of microcystins detected in Ugandan aquatic systems are MC-RR, [Asp^3^]-MC-RY, and [MeAsp^3^]-MC-RY. To date, Lake Saka in Uganda is the only water body with microcystins higher than 60 μg L^−1^ [88].

As a fast-growing economy, Ethiopia generates many domestic and industrial effluents that are changing the physical-chemical conditions and phytoplankton diversity of aquatic ecosystems in the country. Tracking seasonal changes in phytoplankton diversities has revealed the dominance of *Microcystis aeruginosa, M. panniformis, Anabaena spiroides,* and *Cylindrospermopsis* spp. in Ethiopian lakes, which highly correlates with the presence of microcystins (0.58–1547.28 μgL^−1^) [46,73,75,76,116]. The peak concentrations of microcystins in these water bodies are detected during the dry season around November to December of every year, and the main microcystins variants found in lentic ecosystems include MC-LR, MC-YR, MC-RR, MC-dmLR, and MC-LA (Table 3).

### 5.2. North Africa

Studies on cyanobacterial abundance and toxicity in the coastal regions are generally scarce in Africa, making the study by Douma et al. [70] very important. From 2004 to 2013, the authors investigated the diversity of cyanobacteria and the presence of microcystins in a Moroccan coastal lagoon, the Sidi Boughaba, and found that only *Microcystis flos-aquae* produced the microcystin variants MC-LR, MC-RR, and MC-WR (Table 3). Highly toxic microcystin-producing floating and benthic mats of *Nostoc muscorum* are formed from March to October yearly when temperatures are above 10 °C in the Oukaïmeden River [117]. The river is situated in the High-Atlas Mountains of Marrakech, Morocco, representing a critical environment in the country. Characterization of the blooms indicates the production of three microcystin variants, including MC-LR (Table 3).

A high diversity of microcystin variants has been recorded in blooms occurring in Algerian aquatic ecosystems (Table 3). The characterization of *Microcystis* spp. blooms from the Lake des Oiseaux shows the highest diversity of microcystins reported from any African aquatic ecosystem [58]. Twelve (12) out of twenty-one (21) putative congeners are fully characterized (MC-RR, MC-LR, MC-FR, MC-WR, MC-YR, MC-LA, MC-(H4)YR, MC-HilR, [Asp^3^]MC- rAba, [Glu(OCH_3_)^6^]MC-LR), [Asp3]MC-HarAba, and [Glu(OCH_3_)^6^]MC-FR) (Table 3). Among these congeners, the [Asp3]MC-HarAba and [Glu(OCH_3_)^6^]MC-FR are novel congeners found in Algeria. In addition to the high diversity of microcystin variants, Algerian water bodies present some of the highest microcystins found in African aquatic ecosystems (Table 3). Nasri et al. [60], for instance, measured concentrations as high as 26,163 μg L^−1^ in raw lake water, which was equivalent to 4590 μg g^−1^ DW (dry weight) bloom material. Detecting high microcystin concentrations associated with cyano-HABs (cyanobacterial harmful algal blooms) has led to several animal deaths in this country [59]. Bloom extract analyzed by Nasri et al. [59] confirmed that the microcystin variants MC-LR, MC-YR, and MC-RR contributed to the deaths of turtles (*Emys orbicularis* and *Mauremys leprosa*) at Lake Oubeira, Algeria. Autopsies of these terrapins revealed that the highest MC concentrations were detected in the liver, viscera, and muscle tissues.

Most aquatic ecosystems in Egypt, including the Nile River, irrigation canals, lakes, and fish ponds, are plagued with frequent and intense cyanobacterial harmful blooms (cyanoHABs) [118]. In this country, microcystin production has been primarily associated with *Microcystis* blooms. However, blooms containing microcystin-producing *Osciallatoria* spp. reveal microcystin levels ranging from 300 to 877 µg L^−1^ [62,118,119] (Table 3). As in other parts of Africa, MC-LR and MC-RR are the main variants detected in Egyptian waters, and the level of total microcystins approaches ca. 341 µg L^−1^ in the Nile River [118]. Moreover, the levels of microcystins in raw and treated water have frequently been found to exceed the 1.00 µg L^−1^ MC-LR limit set by the WHO [119].

### 5.3. South Africa

South Africa remains one of the regions with the highest investigations on cyanoHABs. In South Africa, most poisonings have been associated with *M. aeruginosa,* and the first case of cattle intoxication by *Microcystis* was reported as far back as 1927 [38]. In the following years, South African cyanobacteria have been characterized by several studies on ecology, toxicology, and water management implications [24,26,38,103,109,120]. 

Eguzozie et al. [103] demonstrated the diversity of microcystin congeners in floating scums of cyanobacteria at the Swartspruit River, South Africa. The main microcystin congeners detected were MC-YR, MC-(H_4_)-YR, MC-LR, (D-Asp^3^, Dha^7^) MC-RR, and MC-RR. Among the variants, MC-LR had the highest concentration (14.10 to 270.7 μg g^−1^ DW), while MC-YR had the lowest (0.15 to 72.28 μg g^−1^ DW).

There are few studies on cyanobacteria and their toxins in Mozambique compared to South Africa [99,100,101]. The country’s poor economic situation is partly responsible for the limited attention given to the presence of microcystins and other cyanobacterial toxins. Few studies have been conducted to determine the presence of microcystins in the public water supply systems of Pequenos Libombos Dam, Nhambavale Lake, and Chòkwé Irrigation Channel of Mozambique. Microcystins were detected in Nhambavale Lake [99,100] and Chòkwé Irrigation Channel [99], reaching ca. 0.10–7.89 μg L^−1^ and 2.1–159.4 ng g^−1^ DW, respectively. MC-LR, MC-YR, and MC-RR produced by *Microcystis* spp. were the most predominant congeners in the studied water bodies.

In Botswana, Phakalane Waste Water Secondary Maturation ponds and the Limpopo River are some water bodies in which microcystins have already been investigated and quantified [40]. MC-RR was highest (53.62 μg g^−1^ DW), followed by MC-LR (12.114 μg g^−1^ DW) in the water bodies. These concentrations differ from what has been reported in other parts of Africa, where MC-LR is frequently the most abundant among microcystin congeners, even considering different aquatic ecosystems.

### 5.4. West Africa

Although comparatively less information exists on the occurrence and distribution of toxic cyanobacterial blooms in West African aquatic ecosystems compared to other parts of Africa, several studies have been conducted to investigate the diversity of potential microcystin producers in the subregion. Kadiri et al. [12] investigated the presence and composition of cyanobacteria coupled with microcystin production in several coastal aquatic ecosystems of Southern Nigeria. Substantial biomasses of *Microcystis aeruginosa* and *Planktothrix aghardii* were found in almost all investigated aquatic ecosystems. Furthermore, *Anabaena* sp. was observed in some of the ecosystems. Microcystin concentrations ranging from 0.19 µg/L in the Eleme River to 7.75 µg/L in Lekki were reported in the study. In 2008, a survey of several aquatic ecosystems in Zaria, Northern Nigeria, recorded potential microcystin producers such as *Anabaena* spp. and *Microcystis* spp. [121]. Other cyanobacterial genera detected during the study included *Spirulina* spp., *Gloetrichia* spp., and *Cylindrospermopsis* spp. Although microcystin levels were below detection limits in some of the surveyed water bodies, some had total microcystins’ concentrations reaching 3.8 µg/L [104]. Another study of aquaculture ponds in Zaria found *Microcystis* spp., *Nostoc*, *Planktothrix,* and *Anabaena* [93]. In this study, total microcystins’ concentrations ranging from 0.6 to 5.89 µg/L were reported in some fishponds. 

In Kano, a city in North West Nigeria and the capital of Kano State, microcystin congeners produced by *M. aeruginosa* isolated from 6 burrow pits were characterized monthly over three years, and four congeners were identified (MC-RR [24%], MC-YR [26%], MC-LR [48%], and MC-LF [2%]) [95]. They found variations in detected microcystin congeners, except for MC-LR, which was found in all samples. Chia and Kwaghe [104] reported variations in the total microcystin content of surface water supply reservoirs in Zaria. In most reservoirs, higher total microcystin concentrations (1.68–3.94 µg/L) were reported during the dry season than in the rainy season. In addition, there was a significant positive correlation between the biomass of *Microcystis aeruginosa* and *Anabaena subcylindrica* with particulate and dissolved microcystin concentrations [104]. 

In Ghana, a study conducted by Addico et al. [89] to identify the cyanobacteria in the Weija and Kpong reservoirs, which are two crucial freshwater sources in the Southern and Eastern parts of the capital Accra, respectively, identified four potentially cyanotoxin-producing genera: *Anabaena flos-aquae, Cylindrospermopsis raciborskii, Microcystis aeruginosa,* and *Planktothrix agardhii.* The phytoplankton biomass comprised 70–90% of these four genera at all water treatment stages. A microcystin analysis from both reservoirs revealed the presence of 6 different unidentified variants and MC-RR in the Weija and Kpong reservoirs, respectively. Microcystin concentrations ranged from 0.03 µg/L in the Kpong reservoir to 3.21 µg/L in the Weija reservoir [89].

Addico et al. [122] conducted an additional study to identify the toxins associated with a strain of *Anabaena flos-aquae,* one of the dominant species found in major Ghanaian freshwater reservoirs. They reported MC-RR as the main microcystin produced, with a concentration of 10.6 µg/g DW. Another study by Addico et al. [90] from water samples of the Brimsu and Kwanyarko reservoirs in the Central Ghana region indicated the presence of the congeners MC-LR, MC-YR, MC-RR, and MC-LA, with concentrations ranging from 0.1 µg/L to 0.79 µg/L at various stages of water treatment. This report was the first MC-LA in Ghana, and *M. aeruginosa* and *Planktothrix agardhii* were the major species found in the reservoirs.

The occurrence of potential microcystin producers has been scarcely documented in other West African countries. In South East Cote d’Ivoire, Kouassi et al. [123] conducted monthly investigations on the cyanobacterial flora of Adzope reservoir for a year. The authors reported *Planktothrix rubescens* and *M. aeruginosa* as predominant species throughout the study. Although microcystin production from either species was not characterized in the study, this revealed the presence of potentially toxin-producing species in the country.

The absence of studies in other African countries does not mean the lack of a rich diversity of cyanobacteria and their metabolites. A close look at phytoplankton ecological studies in several countries confirmed the presence of potential toxin-producing cyanobacteria and the frequent occurrence of cyanobacterial blooms. This absence of data on microcystin congeners and other cyanotoxins highlights a severe problem requiring research and the development of accessible and cost-effective means of evaluating the risk of cyanobacteria and communicating cyanobacterial-related information in the countries. The major limitation is the lack of skilled personnel and researchers in this critical environmental and public health science area.

## 6. Relationship between Genetic Characterization and Geographical Origin within Toxic Cyanobacteria Strains across Africa

Although DNA-based molecular techniques help reveal the presence of genes involved in toxin production, only RNA-based methods (e.g., RT-qPCR, reverse transcription polymerase chain reaction, and transcriptomic approaches) allow the detection of potential toxin-producing cyanobacteria that are actively transcribing the toxin genes into a toxin. Thus, the presence of toxin-producing genes only in cyanobacteria strains does not directly translate to a toxicological threat. Furthermore, toxin genes can be mutated, which prevents or diminishes their transcription and subsequent toxin production [124,125]. 

In a South African study conducted by Ballot et al. [26], the *mcy*E gene of the microcystin gene cluster was found in a microcystin-producing *Microcystis* strain (AB2011/53) but also in eight non-microcystin-producing *Microcystis* strains. Non-toxic cyanobacteria strains may result from significant gene deletion events induced by a transposable element [125]. Surprisingly, an unusual, isolated strain of *Cylindrospermopsis raciborskii* (CYL-BM-07) from Tunisia was identified as a non-microcystin producer, despite the positive results for two segments (*mcy*A and *mcy*E genes) of the microcystin synthetase *mcy* cluster [56]. This result was further confirmed by HPLC and MALDI-TOF analyses. Various mechanisms, including horizontal gene transfer (HGT), mutations, insertions, and deletions, have been proposed to explain non-toxin-producing cyanobacteria possessing parts of toxin-encoding gene clusters [125], which might have led to the loss of toxin production. As stated previously, a phylogenetic analysis of 16S rRNA, ITS-L, and ITS-S sequences indicated a high genotype similarity among African cyanobacterial strains and field samples compared to those from other regions of the world [31,56]. In addition, Tunisian strains clustered together, and their genetic variability differed considerably from different African strains [56]. However, among the four Tunisian *C. raciborskii* strains isolated from the Bir M’cherga Dam tested for the presence of the genes encoding for the hepatotoxin microcystin, only *Cylindrospermopsis raciborskii* strain CYL-BM-07 was positive for *mc*yA and *mcy*E genes. A BLAST search showed that the *mcy*A and *mcy*E genes of CYL-BM-07 were genetically more similar to homologous sequences found in *Microcystis* sp. than those found in other cyanobacteria genera [56], which might be associated with a possible HGT (horizontal gene transfer) mechanism. Therefore, phylogeny, genetic diversity, and geographical distribution play a vital role in determining the ecotoxicological risks that potentially microcystin-producing cyanobacteria pose to environmental and animal health and should be carefully evaluated.

## 7. Conclusions

Molecular evidence of strong genotypic divergences among West, South, East, and North African strains of cyanobacteria were extensively reviewed. These strains possessed distinct genotypes when 16S rRNA and ITS sequences were employed as molecular markers. Despite positive results of microcystin-producing genes (e.g., *mcy*A, -B, -C, -D, -E, and -G) in African strains, this does not necessarily translate to toxin production. It is, however, pertinent to note that the discussed methods were based mainly on the microscopy and Sanger sequencing of PCR products, which limit the knowledge of African cyanobacteria compared to cyanobacterial studies performed in other parts of the world. Whenever possible, metagenomic, metatranscriptomic, and metabolomic approaches should be employed to investigate cyanobacteria from different African regions.

## Figures and Tables

**Table 2:** The *mcy* operon involved in the biosynthesis of microcystins by cyanobacteria.

Gene	Protein	Functions
*mcy*H	McyH	ABC transporter
*mcy*I	McyI	Putative dehydrogenase
*mcy*F	McyF	Amino acid racemase
*mcy*E	McyE	NRPS-PKS (KS-AT-ACP-AMT-C-A-PCP-C)
*mcy*D	McyD	PKS (KS-DH-CM-KR-ACP-KS-AT-DH-KR-ACP)
*mcy*G	McyG	NRPS-PKS (A-PCP-KS-AT-CM-KR-ACP)
*mcy*A	McyA	NRPS (A-NMT-PCP-C-A-PCP-E)
*mcy*B	McyB	NRPS (C-A-PCP-C-A-PCP)
*mcy*C	McyC	NRPS (C-A-PCP-TE)
*mcy*J	McyJ	*O*-acetyltransferase

*mcy*A-C and *mcy*D-J, putative operons bidirectionally transcribed; NRPS, nonribosomal peptide synthetases; PKS, polyketide synthases; NRPS-PKS, hybrid NRPS/PKS; A-domain, substrate activation; NMT-domain, *N*-methylation; PCP, covalent intermediate tethering; C-domain, condensation of intermediates; E-domain, substrate epimerization; TE-domain, cyclization and release; *O*-acetyltransferase, substrate acetylation; ACP, covalent intermediate tethering; KR-domain, ketoreductase; CM-domain, *C*-methyltransferase; DH-domain, dehydratase; AT-domain, acyltransferase; KS-domain, ketosynthase; AMT-domain, transfer of amino group [9,49,52].

**Table 3:** Diversity of microcystin congeners recorded in different aquatic ecosystems in Africa.

Sub-region	Country	Type of Water Body	Major Toxin-Producing Species	Detected Cyanotoxins	Concentration of Detected Cyanotoxins	Detection Method	References
North Africa	Algeria	Freshwater lakes	*Microcystis* spp.	MC-RR, MC-LR, MC-FR, MC-WR, MC-YR, MC-LA, MC-(H4)YR, MC-HilR, [Asp^3^]MCRAba, [Glu(OCH_3_)^6^]MC-LR, and [Glu(OCH_3_)^6^]MC-FR	4590 μg g^−1^ DW total microcystins	LCMS/MS, HPLC	[58,59,60,61]
	Egypt	Nile river; irrigation canals; upper Egypt fishponds	*Microcystis aeruginosa,* *Oscillatoria tenuis,* *Anabaena subcylindrica,* *Anabaena variables,* *Nostoc spongiaeforme,* *Oscillatoria limnetica,* *Phormidium corium,* *Planktothrix agardhii,* *Anabaena variables,* *Plectonema boryanum,* *Raphidiopsis raciborskii*	Total MCs, MC-LR, MC-RR, MC-YR	300 to 877 µg L^−1^ total microcystins	HPLC, NMR, ESIMS, ESIMS-CID-MS, ELISA	[62,63,64,65,66,67]
	Morocco	Reservoirs, ponds, waste stabilization ponds, and rivers	*Nostoc muscorum*	Total MCs, MC-LR, MC-RR, and MC-WR	229.4 μg/g^−1^ DW	ELISA, HPLC, FAB-MS	[68,69,70,71]
	Tunisia	Dams, reservoirs	*Microcystis* spp., *Oscillatoria tenuis,* *M. aeruginosa, R. raciborskii, Planktothrix agardhii*	MC-LR equivalent MC-LR (MC-FR, MC-RR, MC-YR, and MCWR)	0.008–5.57 μg L^−1^	GC/MS, PP2 inhibition assay	[31,59,72]
East Africa	Ethiopia	Reservoirs and lakes	*Microcystis* spp., *Microcystis aeruginosa,* *Microcystis botrys,* *Microcystis flos-aquae* *Microcystis novacekii,* *Microcystis panniformis,* *Anabaena* spp., *Nostoc* spp., *Raphidiopsis* spp., *Raphidiopsis africana,* *Raphidiopsis. Raciborskii*	MC-LR, MC-YR, MC-RR, MC-dmLR, and MC-LA	0.58- 1547.28 μg L^−1^ total microcystins	ELISA, LC-ESI-MS-MRM, HPLC-DAD	[46,73,74,75,76]
	Kenya	Freshwater lakes and saline lakes	*Microcystis aeruginosa,* *Arthrospira fusiformis,* *Microcystis flos-aquae,* *Phormidium terebriformis,* *Oscillatoria willei,* *Spirulina subsalsa*	MC-LR;MC-RR; MC-LF; MC-YR	1.6–19,800 μg g^−1^ DW	HPLC-PDA, MALDI-TOF, ELISA	[29,34,77,78,79,80,81,82]
	Tanzania	Freshwater lakes	*Microcystis* sp.	MC-RR	0–1.0 μg L^−1^	HPLC-DAD	[83,84]
	Uganda	Freshwater lakes	*Microcystis aeruginosa,* *M. flos-aquae,* *Anabaenopsis* spp., *Aphanizomenon* sp., *Anabaena* sp., *Raphidiopsis raciborskii*	MC-RR, (Asp^3^) MC-RR, MC-YR, (Asp^3^) MC-YR, MC-LR, MC-RY, (Asp^3^) MC-RY	0.02–10.00 μg MC-LR eq./L 0.03 to 144 fg cell^−1^ total microcystins	HPLC-DAD, LC-MS/MS, MALDI-TOF MS	[34,35,36,85,86,87,88]
West Africa	Ghana	Freshwater reservoirs	*Microcystis aeruginosa*	MC-RR	0.03–3.21 μg L^−1^ total microcystins, 0.1–0.79 μg L^−1^, 10.60 μg g^−1^ DW MC-RR	HPLC-UV	[89,90]
	Nigeria	Lakes, reservoirs, lagoons, rivers, ponds, floodplain	*Raphidiopsis* sp., *Microcystis* sp., *Microcystis aeruginosa,* *Microcystis flos-aquae,* *Microcystis wesenbergii,* *Lyngbya* sp.	MCs, MC-LR, MC-RR	0.19–7.75, 3.8, 1.68–3.94, 0.6–5.89 μg L^−1^ total microcystins	ELISA, HPLC-DAD	[12,91,92,93,94,95,96,97]
South Africa	Mozambique	Wastewater treatment ponds, dams, lakes, irrigation channels	*Arthrospira fusiformis,* *Microcystis* spp.	MC-LR, MC-YR, MC-RR	0.10–- 7.89 μg L^−1^ 2.1–159.4 ng g^−1^ DW	LCMS	[98,99,100,101]
	Botswana	Ponds, rivers, and lakes	*Microcystis novacekii,* *Raphidiopsis raciborskii,* *Phormidium* spp., *Planktothrix* spp.	MC-RR, MC-YR, MC-LR, and MC-WR	53.62 μg g^−1^ DW MC-RR; 12.114 μg g^−1^ DW MC-LR	LC-MS/MS	[102,103]
	South Africa	Reservoirs, lakes, and rivers	*Microcystis* spp., *Anabaena* spp., *Microcystis aeruginosa,* *Microcystis flos-aquae,* *Raphidiopsis raciborskii,* *Phormidium* spp., *Planktothrix* spp.	MC-LR, MC-YR, MC-RR, MC-(H_4_)YR and (D-Asp^3^, Dha7) MC-RR; MC-LA, MC-LF	14.10 to 270.7 μg g^−1^ MC-LR; 0.15 to 72.28 μg g^−1^ MC-YR	HPLC-DAD, HPLC-UV, ELISA, LC-MS	[20,24,26,39,53,57,102,103,104,105,106,107,108,109,110,111]
	Zimbabwe	Rivers and lakes	*Microcystis* spp., *Raphidiopsis raciborskii,* *Phormidium* spp., *Planktothrix* spp.	MC-LR	0.2–22.48 μg L^−1^	ELISA, HPLC	[102,112,113,114,115]

DW = dry weight.

## Data Availability

All reported data are referenced in the text.
